# Knowledge, attitude, and practices regarding TB among senior management officials of companies with increased silica dust exposure in Lusaka and Southern provinces of Zambia

**DOI:** 10.3389/fepid.2025.1593046

**Published:** 2025-11-03

**Authors:** L. Mutti, M. Kagujje, D. Siameka, R. Hambwalula, M. Maimbolwa, L. M. Ziko, K. Zimba, N. Kasese-Chanda, R. Chimzizi, A. Mubanga, M. Muyoyeta

**Affiliations:** ^1^ Tuberculosis Department, Center for Infectious Disease Research in Zambia, Lusaka, Zambia; ^2^ Health Office, TB/HIV Division, United States Agency for International Development, Lusaka, Zambia; ^3^ USAID LEAP LOCAL Project, National Tuberculosis and Leprosy Program, Ministry of Health Zambia, Lusaka, Zambia; ^4^ National Tuberculosis and Leprosy Program, Ministry of Health Zambia, Lusaka, Zambia

**Keywords:** tuberculosis, silicosis, workplace, policy, knowledge, attitude, practice, employer

## Abstract

**Introduction:**

A high burden of tuberculosis (TB) complicated by occupational risk factors implies a need for the workplace to develop strategies to reduce workplace incidence of TB.

**Methods:**

We conducted a cross-sectional study to establish the knowledge, attitudes, and practices (KAP) related to TB among senior management officials of manufacturing and construction companies associated with exposure to silica dust. The study was conducted in Lusaka and Southern provinces of Zambia between February and October 2022 using a 28-question multiple-choice self-administered electronic questionnaire. Descriptive statistics were used to determine KAP levels. The total score in KAP was calculated based on correct responses out of a maximum of 17, 7, and 14 points, respectively and categorized into “poor” or “good” using the mean/median. Logistic regression was done to explore the association between characteristics and KAP.

**Results:**

Of 118 participants, 48.3% were aged between 31–40 years, 86.4% were male), and 63.6% represented construction companies. The median/mean KAP scores were 8(IQR 6–10), 3.3 (SD 1.66) and 6.00 (IQR 4–8) respectively. Of the participants, only 47.5% (56/118) had good knowledge, 49.2% (58/118) had good attitudes, and 47.5% (56/118) had good practice scores. Individuals aged over 50 years old, female, and officials from construction companies had higher odds of good knowledge (aOR = 7.8, *p* = 0.027; aOR = 4.70, *p* = 0.016 and aOR = 3.45, *p* = 0.008 respectively) and good attitude (aOR = 14.64, *p* = 0.021; aOR = 6.51, *p* = 0.006 and aOR = 3.90, *p* = 0.006 respectively) Participants working in construction companies had higher odds of good practice (aOR = 2.26, *p* = 0.048).

**Discussion:**

Senior management officials had gaps in knowledge despite having favorable attitudes and practices. Companies must be educated on TB alongside efforts to improve attitudes and practices towards TB in the workplace.

## Introduction

Globally, the highest burden of tuberculosis(TB) is seen in economically productive individuals aged between 15 and 64 years old (1). In Zambia, 85% of the total TB incidence is comprised of individuals aged above 15 years old (1). The high burden of TB within this economically productive age group has both public health and socio-economic implications for low- and middle-income countries like Zambia.

Zambia's major industries include mining, construction, drilling and agriculture, and chemical and fertilizer manufacturing (2). These industries are associated with increased exposure to crystalline silica dust particles (3). Individuals that inhale silica dust over prolonged periods have an increased risk of developing silicosis (4). Individuals with silicosis are three to four times more likely to develop TB than those without silicosis in the same workforce, and the risk of developing TB increases with the severity of silicosis (5). Additionally, exposure to silica even without the development of silicosis also increases the chances of an individual developing TB (6). In addition to the increased risk of progression from infection to disease due to silicosis and silica dust exposure, the poor working conditions in some of the above industries such as overcrowding, and prolonged working hours in areas with poor ventilation also increase the risk of TB infection.

In 2014, the incidence rate of pulmonary TB among miners in Zambia was 288 per 100,000 population (7). While this is lower than the prevalence of TB in the general population (8), this could be attributable to the strict implementation of the occupational health and safety act (9, 10) within formal mining companies which mandates screening for occupational diseases among miners and ex-miners. Alternatively, it may reflect miners seeking care in facilities where their occupation is not documented, as individuals with TB or a history of TB are not permitted to work in the mines under the workers compensation act (11). Beyond the data available from the formal mining sector, there are no data on the burden of TB among individuals exposed to silica dust in other industries, such as small-scale mining, quarrying, and construction. Outside of mining, the current available Zambian workplace policy was developed in 2005 (12) and provides explicit guidance on HIV/AIDS and STDS with little mention on workplace policies for prevention, early identification and support for employees on treatment for TB. This leaves the workplace vulnerable to continued spread of TB amongst employees, their families and the community eventually affecting workplace productivity.

A high burden of TB in Zambia compounded by occupational risk factors underscores the significant need for the Zambian workplace to develop and implement strategies and procedures that reduce workplace risk for TB. This study aimed to determine the knowledge, attitudes, and practices toward TB among senior management officials in the manufacturing, mining and construction industries that are associated with increased exposure of workers to silica dust.

## Methodology

### Study design and setting

We conducted a descriptive cross-sectional study between February 2022 and October 2022 in two provinces of Zambia, specifically Lusaka and Southern Province.

### Population, sampling strategy and sample size

The sample population was generated from the company database of the Zambia Chamber of Commerce and Industry (ZACCI) an umbrella organization representing active businesses across the country and all sectors of the economy. The organization advocates for workplace legislature and a business environment that promotes growth. We selected all registered companies with known silica dust production under the following categories: mining, construction, cement manufacturing, brick and tile manufacturing, and glass and ceramic manufacturing. This included 1 mining company, 73 construction companies, 1 cement manufacturing company, 9 brick and tile manufacturing companies, and 10 glass and ceramic manufacturing companies.

The study targeted senior management officials defined as either the most senior staff of an organization or business, or at least the head of a division or department. At least one company official was requested to fill out the questionnaire, with a maximum of two responses per company.

### Data collection

A multiple-choice e-questionnaire was developed following WHO recommendations for TB KAP surveys (13). The survey was self-administered in English language through Google Forms. A total of 28 closed-ended mandatory questions were developed of which 7 were knowledge questions, 8 were attitude questions and 13 were practice questions. Of the practice questions, 5 questions were included to support TB programming and therefore were not added to the overall analysis of practice scores. A total of 21 questions were multiple choice while 7 had a single best answer option.

The survey was sent out via email. A total of 3 reminders were sent out via email; an initial invite to participate, a reminder to participate 7 days from the initial invite, and a last reminder which was sent out 7 days from the last reminder. Physical follow-ups were planned for companies that would not respond to email invitations. The survey was then completed by all the participants independently on a tablet between 27th June 2022 and 30th November 2022.

### Response validation

Responses for each question within the questionnaire were independently scored by 3 medical doctors. A response was considered correct when at least 2 medical doctors agreed on it.

### Data management and statistical analysis

Responses from each question for each participant were stored on Google Forms. All responses were downloaded onto a.csv file and imported into Python (version 3.8) for data recoding. The file was then imported into STATA (Stata Corporation Version 18.5) for cleaning and analysis.

We performed descriptive statistics to determine the frequency and proportion of participant characteristics and categorical variables.

A scoring system was assigned to summarize knowledge, attitude, and practice responses. Each correct response received a score of 1 and each incorrect response received a score of 0. Responses marked as “don't know,” “Not sure,” or “other” were treated neutrally with a score of 0. Ambiguous “other” responses were excluded from subsequent analyses to ensure precision and clarity.

The total score for each participant in knowledge, attitude, and practice was calculated based on the correct responses out of a maximum of 17, 7, and 14 points, respectively. Categorization into “poor” or “good” knowledge, attitude, and practices was determined using the mean for normally distributed data and the median for data that did not fit within the normal distribution.

Logistic regression analysis was utilized to explore the association between participant characteristics and knowledge, attitude, and practice. We first conducted univariable analysis to identify characteristics associated with knowledge, attitude and practice. Next, a multivariable analysis was conducted to control confounding variables. A statistical cutoff point of 0.2 was used to select variables for inclusion in the multivariable analysis. In the multivariable analysis, variables with the highest *p*-value (more than 0.05) were removed from the model, leaving only those with significant *p*-values (less than 0.05). However, in cases where only two variables were significant in the univariable analysis, the variable with the higher *p*-value (more than 0.05) was retained in the multivariable model.

### Ethics statement

Ethical approval was obtained from the University of Zambia Biomedical Research Ethics Community (UNZA-BREC) (Reference Number: 2327-2021) who approved the use of the electronically documented informed consent. Informed consent was provided as the first section of the electronic questionnaire. The participants could only proceed to complete the questionnaire if consent was granted. The consent has been collected and saved digitally as part of a completed questionnaire and data collection.

## Results

### Company response rate

Of the 94 companies, 85 (90.4%) were successfully contacted, and at least one representative from each provided a response to the questionnaire.

### Participant characteristics

A total of 118 individuals completed the questionnaire (Table 1) giving a response rate of 69.4%. Of the individuals who completed the questionnaire, 48.3% were aged between 31 and 40 years old, and 86.4% were male participants. Of the participants, 47.5% were from operations and logistics department and 63.6% of participants were from Construction companies.

**Table 1:** Participant characteristics (*n* = 118).

Characteristics	Descriptive statistics	
	Frequency *N* = 118	Percentage
Age group (years)		
Under 30	36	30.5
31–40	57	48.3
41–50	16	13.6
Over 50	9	7.6
Gender		
Male	102	86.4
Female	16	13.6
Department		
Chief Executive Officer/Chairman	44	37.3
Finance	2	1.7
Health and safety	2	1.7
Human resource	14	11.9
Operations or logistics	56	47.5
Company category		
Cement and concrete	40	33.9
Ceramic production	3	2.5
Construction	75	63.6

### Knowledge of TB

The median knowledge score was 8 points [Interquartile range (IQR) 6–10]. Overall, 52.5% of participants showed poor knowledge and 47.5% had good knowledge (Table 2).

**Table 2:** Knowledge of TB among study participants.

TB knowledge questions	Knowledge scores			
	Descriptive statistics	Correct response	Incorrect responses	Do not know
	Frequency (%)			
How serious a problem do you think TB is in Zambia		38 (32.2)	80 (67.8.0)	0 (0.0)
Very serious	38 (32.2)			
Somewhat serious	49 (41.5)			
Not very serious	31 (26.3)			
What are the signs and symptoms of TB		328 (86.6)	41 (11.1)	1 (0.3)
Rash	7 (5.9)			
Cough	64 (54.2)			
Cough that lasts >3 weeks	44 (37.3)			
Coughing blood	27 (22.9)			
Severe headaches	17 (14.4)			
Nausea	10 (8.5)			
Weight loss	32 (27.1)			
Fever	21 (17.8)			
Fever without clear cause that lasts more than 7 days	7 (5.9)			
Chest pain	69 (56.3)			
Shortness of breath	50 (42.4)			
Ongoing fatigue	21 (17.8)			
How can a person get TB?		93 (38.1)	132 (54.1)	19 (7.8)
Through handshakes	17 (14.4)			
Through the air when a person with TB coughs or sneezes	93 (78.8)			
Through sharing dishes	10 (8.5)			
Through eating from the same place	10 (8.5)			
Through touching items in public places (doorknobs, handles)	93 (78.8)			
Depends on the genes	1 (0.9)			
Exposure to dust and smoking	1 (0.9)			
How can a person prevent from getting TB		136 (54.4)	100 (40.0)	14 (5.6)
Avoid handshakes	24 (20.3)			
Cover nose and mouth	87 (73.7)			
Avoid sharing dishes	15 (12.7)			
Wash hands after touching items in public places	37 (31.4)			
Being in well-ventilated places	34 (28.8)			
Through good nutrition	24 (20.3)			
Taking medicine to prevent TB	15 (12.7)			
Who can have TB?		100 (100.0)	0 (0)	0 (0.0)
Anybody	105 (89.0)			
Poor people	2 (1.7)			
Homeless people	2 (1.7)			
Alcoholics	8 (6.8)			
Drug users	7 (5.9)			
People living with HIV/AIDS	14 (11.6)			
People who inhale dust which has silica	14 (11.6)			
Can TB be cured?		98 (83.1)	1 (0.8)	19 (16.1)
Yes	98 (83.1)			
No	1 (0.9)			
If you think TB can be cured, how can it be cured?		87 (77.7)	20 (17.9)	5 (4.2)
Herbal remedies	12 (10.2)			
Home rest without medicine	2 (1.7)			
Praying and fasting	2 (1.2)			
Specific drugs given by health centre	87 (73.7)			
Any antibiotics available over the counter at any pharmacy	4 (3.4)			
Overall knowledge result		Median Score = *8 points (IQR 6–10)*		
		Good Knowledge = 4*7.5% (56/118)*		
		Poor knowledge = 5*2.5% (62/118)*		

In terms of the burden of TB, 32.2% of participants thought TB is a serious problem in Zambia. Overall, 38.1% of participants knew that TB is transmitted “through the air when a person with TB coughs or sneezes” while 54.1% selected incorrect responses including touching items in public places, handshakes, and sharing dishes.

Covering the nose and mouth (73.7%) and being in well-ventilated places (28.8%) were correctly selected as modes to prevent TB spread. Additionally, taking medicine to prevent TB (12.7%) was identified as a way a person could prevent getting TB.

In terms of TB symptoms, 86.6% of participants selected correct TB symptoms. Poor knowledge of TB symptoms was identified in 11.1% of incorrect responses that included rash (5.9%), nausea (8.5%), shortness of breath (42.4%), and ongoing fatigue (17.8%).

Generally, 83.1% of participants knew TB is curable, and is treated with specific drugs provided by the health facility. Of the incorrect responses, 10.2% reported TB being cured using herbal medication, and 3.4% with any antibiotics available over the counter at any pharmacy.

### Attitude towards TB

The mean attitude score was 3.3 points [Standard Deviation (SD) 1.66]. Overall, 50.9% of participants had poor attitudes. (Table 3).

**Table 3:** Attitude towards TB among study participant**s.**

TB questions on attitude	Attitude scores			
	Descriptive statistics	Correct responses	Incorrect responses	Do not know
	Frequency (%)			
Do you think this company should be concerned about TB		83 (70.3)	35 (29.7)	0 (0.0)
Yes	83 (70.3)			
No	35 (29.7)			
Why do you think this company should be concerned about TB		140 (87.5)	20 (12.5)	0 (0.0)
Exposure to Silica dust	41 (34.8)			
Poor respiratory hygiene	8 (6.8)			
Poor ventilation	3 (2.5)			
No personal protective equipment	9 (7.6)			
Because we are all at risk of TB whether at work or home	49 (41.5)			
Affects employee productivity	24 (20.3)			
It can cause employee absenteeism	26 (22.0)			
Too much dust	0 (0.0)			
Why do you think this company should not be concerned about TB		0 (0)	55 (100)	0 (0.0)
TB does not affect individuals at this company	9 (7.6)			
There is good ventilation at our company	18 (15.3)			
The company practices respiratory hygiene	10 (8.5)			
PPE is provided	17 (14.4)			
Workplace policy in place already	1 (0.9)			
Do any of your employees have TB or had TB in the past^a^				
Yes	6 (5.1)			
No	66 (55.9)			
Which statement is closest to your feelings about people with TB		74 (63.2)	43 (36.8)	0 (0.0)
I feel compassion and desire to help	74 (62.7)			
I feel compassion but I tend to stay away from these people	21 (17.8)			
It is their problem, and I cannot get TB	0 (0.0)			
I fear them because they may infect me	4 (3.4)			
I have no particular feeling	18 (15.3)			
Other	1 (0.9)			
In your workplace how would a person who has TB be regarded/treated		82 (73.9)	29 (26.1)	0 (0.0)
Most people reject him/her	3 (2.5)			
Most people are friendly, but they generally try to avoid him or her	26 (22.0)			
The workplace mostly supports him or her	82 (69.5)			
Other	7 (5.9)			
How would you treat a person you work with who has had TB		0 (0.0)	106 (100.0)	0 (0.0)
Keep away from them	9 (7.6)			
Do not talk to them	0 (0.0)			
Do not sit next to them	4 (3.4)			
Nothing as they should be at home during treatment	93 (78.8)			
Other	12 (710.2)			
How expensive do you think TB diagnosis and treatment is in this county		2 (2.4)	26 (30.6)	57 (67.1)
It is free of charge	2 (1.7)			
It is reasonably priced	20 (17.0)			
It is somewhat/ moderately expensive	4 (3.4)			
It is very expensive	2 (1.7)			
Overall Attitude Result		Mean Score = *3.33 points SD 1.66*		
		Good attitude = 49.2*% (58/118)*		
		Poor attitude = 50.9*% (60/118)*		

SD, standard deviation; ^a^Question not scored.

The most reported reasons for why the company should be concerned about TB were exposure to silica dust (34.8%), the effect on employee productivity (20.3%), its potential to cause employee absenteeism (22.0%), poor respiratory hygiene (6.8%), lack of personal protective equipment (PPE) (7.6%) and poor ventilation (2.5%). Participants reported no concern towards TB due to good ventilation (15.3%).

Most participants felt the desire to help individuals with TB in the workplace (62.7%), while 36.8% of participants felt compassion but kept a distance or feared due to the possibility of infection transmission or had no feeling at all. In addition, 78.8% of participants stated they would not treat individuals with TB in any way as individuals with TB are expected to be at home not the workplace. The survey found that individuals with TB would be treated in a friendly manner but generally avoided by co-workers (22.0%). Supporting the individual with TB in the workplace was reported in 69.5% of individuals.

### Practices related to TB

The median practice score was 6 points (IQR4–8). Overall, good practices were identified in 47.5% of participants. As per Table 4, of the total participants, 15.3% reported having a workplace policy in place, while of those who did not have a workplace policy, 92.6% were willing to develop a TB workplace policy. Majority participants recommended the inclusion of prevention activities (52.5%), TB awareness and sensitization (48.3%), provision of PPE (44.9%), TB screening (40.7%), health and safety (29.7%), improved ventilation (18.6%), psycho-social support (14.4%), anti-TB stigma and discrimination policy (11.0%), full pay sick leave (9.3%), half pay sick leave (12.7%), or unpaid sick leave but with guarantee job continuity once stable to work (11.9%).

**Table 4:** TB practices among study participants.

Questions on practices	Practices scores			
	Descriptive statistics	Correct responses	Incorrect responses	Do not know
	Frequency (%)			
Does this company have a policy in place that focuses on workplace TB prevention, control, and management		18 (15.3)	81 (68.6)	19 (16.1)
Yes	18 (15.3)			
No	81 (68.6)			
If your company does not have a TB workplace policy, are you willing to develop such a policy		75 (92.6)	6 (7.4)	0 (0.0)
Yes	75 (92.6)			
No	6 (7.4)			
If you are not willing to develop a workplace policy, kindly select reasons why		0 (0)	2 (33.3)	4 (66.7)
TB is not an important problem	1 (16.7)			
No funding for TB	1 (16.7)			
If you are willing to develop a workplace policy, what type of recommendations would you include in it		301 (86.7)	46 (13.3)	0 (0.0)
Prevention activities	62 (52.5)			
Health and Safety	35 (29.7)			
TB screening	48 (40.7)			
TB awareness and sensitization activities	57 (48.3)			
Anti-TB stigma and discrimination policy	13 (11.0)			
Pyscho-social support during treatment	17 (14.4)			
Fully paid sick leave	11 (9.3)			
Half-pay sick leave	15 (12.7)			
Unpaid sick leave but with guarantee of job continuity once stable to work	14 (11.9)			
Improving ventilation	22 (18.6)			
Provision of personal protective equipment	53 (44.9)			
Other	5 (4.2)			
What does the company do to provide information to its employees about TB		35 (29.7)	69 (58.5)	14 (11.9)
Health talks on TB provided by peer educators	16 (13.6)			
Health talks on TB provided by trained health personnel	15 (12.7)			
Correspondence via email, brochures on TB	3 (2.5)			
Short videos on TB	0 (0.0)			
Short plays on TB	1 (0.9)			
No information about TB	69 (58.5)			
Which of the following practices in your workplace prevent the spread of TB		151 (95.6)	7 (4.4)	0 (0.0)
Open windows	49 (41.5)			
Encouraging individuals to cover their nose and mouth with tissue when they cough and/or sneeze	87 (73.7)			
Terminate contract of all employees identified as TB patients	4 (3.4)			
Isolate all employees with TB in one office space	3 (2.5)			
Actively screening for TB during the year	15 (12.7)			
What does the company do to help identify employees with TB		16 (13.8)	100 (86.2)	0 (0.0)
Nothing—wait for employee to announce their diagnosis	83 (70.3)			
Mandatory screening during the year	16 (13.6)			
Optional screening during the year	17 (14.4)			
When someone is found with TB what is the workplace next course of action?		77 (66.4)	39 (33.6)	0 (0.0)
Terminate contract	3 (2.5)			
Allow sick leave for 1 month then terminate contract	9 (7.6)			
Allow sick leave for the duration of the illness	77 (65.3)			
Allow unpaid leave for the duration of illness	3 (2.5)			
Allow half pay leave for duration of illness	24 (20.3)			
Does this company have an onsite clinic or sick bay for individuals who fall ill^a^				
Yes	11 (9.3)			
No	96 (81.4)			
Maybe	11 (9.3)			
If this company has an onsite clinic or sick bay for individuals who fall ill, what services are available at that facility^a^				
Prevention services	10 (8.5)			
TB screening services including sputum collection	4 (3.4)			
Diagnostic services including sputum analysis by Gene Xpert	1 (0.9)			
Diagnostic services including sputum analysis by microscopy	3 (2.5)			
TB treatment services including drug dispensation	3 (2.5)			
TB treatment services including treatment monitoring	3 (2.5)			
No TB services are provided	84 (71.2)			
I don't know which services are provided	21 (17.8)			
If this company has an onsite clinic for individuals who fall ill have the providers been trained in TB management^a^				
Yes	6 (5.1)			
No	90 (76.3)			
I don't know	22 (18.6)			
Does this company have a budget that goes towards health care of employees^a^				
Yes	88 (74.6)			
No	16 (13.6)			
I don't know	14 (11.9)			
If this company has a budget towards healthcare of employees, what percentage of the budget is place toward health care^a^				
0%–20%	67 (56.8)			
20%–40%	5 (4.2)			
40%–60%	2 (1.7)			
60%–80%	0 (0.0)			
80–100%	1 (0.9)			
I don't know	43 (36.4)			
Overall practice	Median score = 6 *points (IQR 4–8)*			
	Good practice = 47.5*% (56/118)*			
	Poor practice = 52.5*% (62/118)*			

IQR, interquartile range, ^a^Programmatic question (not scored).

In terms of the workplace providing information on TB, participants reported that 58.5% of companies did not provide any information on TB.

In terms of practices to prevent the spread of TB, participants reported encouraging individuals to cover their nose and mouth when they cough or sneeze (73.7%), and actively screen for TB during the year (12.7%).

To assist in the identification of TB in employees, 13.8% of participants responded to mandatory or optional screening. Of those individuals found with TB in the workplace, 65.3% of participants selected to allow sick leave for the duration of illness.

Of the total responses, 74.6% of participants reported having a budget towards the healthcare of employees ranging between 0%–20% of the total budget (56.8%).

### Regression analysis

In the univariable analysis participants from construction companies had a higher odds of good knowledge of TB [cOR 2.97 (1.31- 6.71) *p*-value 0.009]. With regards to attitude, individuals aged over 50 years, females and participants from Human resource departments and construction companies had higher odds of good attitude towards TB (cOR8.94 (1.01–79.05) *p*-value 0.49, cOR 3.65 (1.10–12.09) *p*-value 0.034, cOR 6.42 (1.56–26.46) *p*-value 0.010 and cOR 2.64 (1.18–5.90) *p*-value 0.018 respectively) (Table 5).

**Table 5:** Univariable logistic regression analysis of KAP among study participants.

Characteristics	Knowledge		Attitude		Practices	
	COR (95% CI)	*p*-value	COR (95% CI)	*p*-value	COR (95% CI)	*p*-value
Age group (years)						
Under 30	Ref.	Ref.	Ref.	Ref.	Ref.	Ref.
31–40	1.32 (0.56–3.08)	0.524	0.76 (0.34–1.75)	0.515	1.45 (0.62–3.36)	0.387
41–50	2.02 (0.61–6.67)	0.248	1.86 (0.56–6.22)	0.312	0.84 (0.25–2.82)	0.778
Over 50	5.5 (1.00–30.36)	0.050	8.94 (1.01–79.05)	**0.049**	2.8 (0.60–13.01)	0.189
Gender						
Male	Ref.	Ref.	Ref.	Ref.	Ref.	Ref.
Female	2.79 (0.90–8.60)	0.075	3.65 (1.10–12.09)	**0.034**	2.03 (0.69–6.00)	0.201
Department						
CEO/Chairman	Ref.	Ref.	Ref.	Ref.	Ref.	Ref.
Finance	–	–	–	–	–	–
Health and safety	–	–	–	–	1.75 (0.10–29.92)	0.699
Human resource	1.93 (0.57–6.51)	0.291	6.42 (1.56–26.46)	**0.010**	3.15 (0.90–11.04)	0.073
Operations or logistics	1.25 (0.56–2.78)	0.581	1.63 (0.73–3.65)	0.236	1.75 (0.78–3.92)	0.174
Company category						
Cement and concrete	Ref.	Ref.	Ref.	Ref.	Ref.	Ref.
Ceramic production	4.67 (0.39–56.51)	0.226	–	–	3.71 (0.31–44.66)	0.301
Construction	2.97 (1.31–6.71)	**0.009**	2.64 (1.18–5.90)	**0.018**	2.12 (0.96–4.69)	0.063

COR, crude odds ratio; CI, confidence interval; CEO, chief executive officer.

Bold indicates *p*-value <0.005.

Using multivariable logistic regression analysis good knowledge was identified in individuals aged over 50 years old [adjusted Odds Ratio (aOR) 7.80 (1.27–48.00) *p*-value 0.027], females [aOR 4.70 (1.33–16.58) *p*-value 0.016], and officials from construction companies [aOR 3.45 (1.39–8.56) *p*-value 0.008]. Individuals aged over 50 years old, females and officials from construction companies had significantly good attitudes towards TB [aOR 14.64 (1.50–143.17) *p*-value 0.021, aOR 6.51 (1.71–24.69) *p*-value 0.006, and aOR 3.90 (1.49–10.21) *p*-value 0.006 respectively] (Table 6). Finally, only participants from construction companies had significantly good practice regarding TB (aOR 2.26 (1.02–5.08) *p*-value 0.048 (Table 6).

**Table 6:** Multivariable logistic regression analysis of KAP among study participants.

Characteristics	Knowledge		Attitude		Practices	
	aOR (95% CI)	*p*-value	aOR (95% CI)	*p*-value	aOR (95% CI)	*p*-value
Age group (years)						
Under 30	Ref.	Ref.	Ref.	Ref.	Ref.	Ref.
31–40	1.26 (0.48–3.28)	0.640	0.70 (0.26–1.87)	0.475	–	–
41–50	2.22 (0.59–8.31)	0.235	2.26 (0.59–8.71)	0.237	–	–
Over 50	7.80 (1.27–48.00)	**0** **.** **027**	14.64 (1.50–143.17)	**0.021**	–	–
Gender						
Male	Ref.	Ref.	Ref.	Ref.	Ref.	Ref.
Female	4.70 (1.33–16.58)	**0** **.** **016**	6.51 (1.71–24.69)	**0.006**	2.34 (0.77–7.16)	0.136
Company category						
Cement and concrete	Ref.	Ref.	Ref.	Ref.	Ref.	Ref.
Ceramic production	8.62 (0.67–110.50)	0.098	–	–	4.36 (0.36–53.06)	–
Construction	3.45 (1.39–8.56)	**0** **.** **008**	3.90 (1.49–10.21)	**0.006**	2.26 (1.02–5.08	**0.048**

aOR, adjusted odds ratio; CI, confidence interval; CEO, chief executive officer.

Bold indicates *p*-value <0.005.

## Discussion

In this study, which involved 118 participants from 85 companies with increased silica exposure in Lusaka and Southern province of Zambia, we report poor knowledge, attitude and practices towards tuberculosis among senior management in the workplace. Within the knowledge section, participants showed poor knowledge in understanding the national burden of TB and modes of TB transmission. Participants over 50 years of age, females and officials from construction companies had higher odds of good knowledge. Individuals over 50 years, females and participants from construction companies had higher odds of a good attitude, however overall poor attitude was identified particularly highlighting the stigma associated with tuberculosis. Poor practices were highlighted in areas concerning workplace screening and the provision of information about TB. Participants from constructions companies showed higher odds of good practices.

The study was able to identify misconceptions related to TB prevention and management as a proportion of participants reported TB was spread by touching public places and could be cured using herbal remedies. This was similar findings from a study in Bangladesh and Zimbabwe which showed manufacturing workers had misconceptions about TB prevention and management (14, 15). Much like other studies (16, 17), this study revealed stigmatizing attitudes by senior-level officials towards individuals with TB which impacts employee willingness to accept symptoms, seek treatment, and/or accept diagnosis. Good knowledge and attitude toward TB in elderly individuals found in this study is similar to a study in Myanmar that looked at determinants of correct knowledge of TB (18). Our study showed that the main reason companies need to be concerned about TB is the high exposure to silica dust. This highlighted that participants had adequate knowledge about silica dust increasing the risk of TB which is different from other studies where knowledge levels of silica and silicosis were low (19, 20). A KAP study done in Zambia (21) showed high knowledge of TB, which is different from our overall poor knowledge result. This, however, does not place uncertainty on our results as the study participant focus was mining employees at direct risk of TB while our study focus is on senior management officials who have a role in policy and oversight. A KAP study by Moyo et al. in Zimbabwe found that effective control of TB in artisanal and small-scale miners is the provision of PPE and access to health care services (15). This is similar to our study where participants recommended improved access to PPE for TB prevention and workplace screening for TB.

The low KAP scores in our study highlight a significant gap in TB programming in the workplace. These low scores could be attributed to the lack of a workplace policy specifically for TB which would guide companies where employees are at higher risk of developing TB. This study adds to the body of increasing evidence and a call to the need for a TB workplace policy in Zambia (4, 7, 21). The low KAP scores underpin the importance of close collaboration of National TB programs with other ministerial bodies to increase sensitization and awareness on prevention and screening of high-risk employees.

The misconceptions surrounding TB prevention in this study could be related to the non-pharmaceutical intervention (NPI) measures for COVID-19. The COVID-19 pandemic significantly reduced the incidence of other respiratory infections (22, 23) and slowed the global achievements toward the END TB strategy (1). Despite this negative impact on progress toward the End TB strategy, studies have shown that COVID-19 NPI measures have been associated with a positive reduction in hospitalization for other acute respiratory infections in children (24) and reduced exacerbation of chronic obstructive pulmonary diseases (25). National TB programs could use the COVID-19 NPI strategies as a blueprint for multi-level social mobilization and communication to disseminate information on TB to communities and workplaces.

A major bottleneck in TB practices identified in this study was that companies did not provide information about TB to their employees, make an effort to help identify employees with TB, and companies were inclined to terminate the contracts of individuals with TB. These bottlenecks have significant implications for TB control regarding prevention and case detection in an age group that carries most of the TB burden. In a country that experiences high TB death rates due to late diagnosis (26), poor health-seeking behaviors (27) are further perpetuated by the thought of loss of income from being diagnosed with TB. This could further lead to delayed presentation at health facilities and/or non-adherence and missed drug pick-ups. Both of which would impact successful TB treatment outcomes for these economically productive individuals.

The End TB strategy milestone on catastrophic costs clearly states that “No TB patient and their households face catastrophic costs as a result of TB” (28). This study shows that economically productive individuals could face severe financial ramifications as senior management officials prefer to provide either half-pay or unpaid sick leave for individuals with TB. This could contribute to the failure to achieve a crucial milestone in the END TB strategy as a nation, as 58% of TB patients in Zambia face catastrophic costs (29). Although participants would allow sick leave for the duration of TB illness for an employee, it also has significant ramifications for company productivity and outputs. A workplace policy must be put in place that benefits both the employee with TB and the company without costly effects on both.

On one hand, a major strength of this study is that it included participants who were registered members of an umbrella organization representing registered and active businesses across the country advocating for a business environment that promotes business growth and pioneers in policy development. The study was able to capture useful data to guide TB workplace policy development. Additionally, it was able to identify potential resources that could be used to support workplace TB initiatives and aid national programs in leveraging locally sourced finances to combat TB.

On the other hand, a limitation of only having members registered from ZACCI in only two provinces potentially introduces selection bias in the study and results cannot be generalized; thus the validity of results cannot be confirmed for all companies in Zambia with high silica exposure. Additionally, reporting bias could have been introduced as few questions required the senior management participants to have medical information that could be deemed private for employees. With high levels of stigma and fears of loss of income chances are the employees do not report TB illness to senior management (21). Lastly, the study was also limited by a small sample size.

## Conclusion

To conclude, this study revealed that most senior management officials in companies with increased silica exposure had poor knowledge, attitude and practices of TB with regards to prevention care and treatment. Although available legislature implies protection for employees in Zambia regarding TB, there is need for National TB programs to leverage the willingness of these companies to identify and treat individuals with TB working in high-risk environments with silica exposure apart from mining. The significant gaps in knowledge, attitude and practices in silica exposed companies underscores the need for Zambia to develop a TB specific workplace policy that covers key working populations in a bid for multisectoral TB epidemic control.

## Data Availability

The raw data supporting the conclusions of this article will be made available by the authors, without undue reservation.
